# Identification of Cytoprotective Small-Molecule Inducers of Heme-Oxygenase-1

**DOI:** 10.3390/antiox11101888

**Published:** 2022-09-23

**Authors:** Gelare Ghajar-Rahimi, Amie M. Traylor, Bini Mathew, James R. Bostwick, N Miranda Nebane, Anna A. Zmijewska, Stephanie K. Esman, Saakshi Thukral, Ling Zhai, Vijaya Sambandam, Rita M. Cowell, Mark J. Suto, James F. George, Corinne E. Augelli-Szafran, Anupam Agarwal

**Affiliations:** 1 Department of Medicine, University of Alabama at Birmingham, Birmingham, AL 35233, USA; 2 Nephrology Research and Training Center, University of Alabama at Birmingham, Birmingham, AL 35233, USA; 3 Division of Cardiothoracic Surgery, Department of Surgery, University of Alabama at Birmingham, Birmingham, AL 35233, USA; 4 Southern Research, Birmingham, AL 35205, USA; 5 Department of Cell, Developmental and Integrative Biology, University of Alabama at Birmingham, Birmingham, AL 35233, USA; 6 Department of Veterans Affairs, Birmingham, AL 35233, USA

**Keywords:** acute kidney injury, heme oxygenase, cisplatin nephrotoxicity, small-molecule drugs, high-throughput screen

## Abstract

Acute kidney injury (AKI) is a major public health concern with significant morbidity and mortality and no current treatments beyond supportive care and dialysis. Preclinical studies have suggested that heme-oxygenase-1 (HO-1), an enzyme that catalyzes the breakdown of heme, has promise as a potential therapeutic target for AKI. Clinical trials involving HO-1 products (biliverdin, carbon monoxide, and iron), however, have not progressed beyond the Phase ½ level. We identified small-molecule inducers of HO-1 that enable us to exploit the full therapeutic potential of HO-1, the combination of its products, and yet-undefined effects of the enzyme system. Through cell-based, high-throughput screens for induction of HO-1 driven by the human HO-1 promoter/enhancer, we identified two novel small molecules and broxaldine (an FDA-approved drug) for further consideration as candidate compounds exhibiting an E_max_ ≥70% of 5 µM hemin and EC_50_ <10 µM. RNA sequencing identified shared binding motifs to NRF2, a transcription factor known to regulate antioxidant genes, including *HMOX1*. In vitro, the cytoprotective function of the candidates was assessed against cisplatin-induced cytotoxicity and apoptosis. In vivo, delivery of a candidate compound induced HO-1 expression in the kidneys of mice. This study serves as the basis for further development of small-molecule HO-1 inducers as preventative or therapeutic interventions for a variety of pathologies, including AKI.

## 1. Introduction

Heme oxygenase-1 (HO-1) is a 32 kDa microsomal enzyme responsible for catalyzing the rate-limiting step of heme degradation into equimolar amounts of biliverdin, iron, and carbon monoxide (CO) [1,2,3]. Ubiquitously distributed in mammalian tissues, HO-1 is induced by a variety of stressors, including heme, growth factors, hypoxia, cytokines, and heavy metals. HO-1 induction confers protection through regulation of inflammation, oxidative stress, apoptosis and autophagy [4], myeloid cell migration [5], and immune cell signaling in several pathologies including nonalcoholic fatty liver disease [6], lung disease [7], and acute kidney injury (AKI) [8]. In the kidney, HO-1 has been implicated in both renal and postrenal forms of AKI, as well as the transition from AKI to chronic kidney disease (CKD) [9,10]. Novel therapeutic options for AKI hold enormous potential for impacting patient health and the healthcare costs resulting from AKI, which affects nearly 20% of adults and 33% of children in the hospital [11]. AKI is associated with significant morbidity and mortality and has no current treatment options beyond supportive care and dialysis. 

Substantial preclinical evidence supports the rationale for developing therapeutic interventions that modulate HO-1 in kidney injury (reviewed by Nath et al. [3]). Past and ongoing clinical trials have focused on targeting the direct or downstream products of the HO-1 pathway (e.g., NCT00531856), but these trials have not been encouraging and, thus, have not progressed beyond Phase ½. Recent data also suggest that, in addition to potential nonspecific effects and toxicities, targeting just one of the products of the HO-1 system may not be adequate for protection, and targeting a combination of two or all three of the byproducts, namely, CO, iron (via ferritin), and biliverdin, may be necessary for optimal benefit. In addition, while the beneficial effects of HO-1 are ascribed to its products, there is still an accepted view that yet-undefined effects of HO-1 induction account for its protective properties. Thus, there is a great clinical need for therapeutic strategies that work through direct induction of endogenous HO-1 rather than administering its products singly or in combination. 

Here, we conducted a cell-based high-throughput screen to facilitate the discovery of small-molecule inducers of endogenous HO-1. We identified SRI-37618 as a potent inducer of HO-1 that is capable of acting through the human *HMOX1* promoter/enhancer, protecting against cisplatin-induced apoptotic cell death in vitro, and inducing HO-1 in vivo in the kidney when delivered intraperitoneally in mice. This work serves as the foundation on which candidate small-molecule inducers of HO-1 will be optimized for further preclinical development and subsequent clinical translation.

## 2. Materials and Methods

### 2.1. Stable Cell Lines

Two stable cell lines containing a human HO-1 promoter/enhancer–reporter construct (4.5 kb + 220 bp HO-1-Luc cells) and a construct with triple mutations in the HO-1 promoter (Mut4.5kb HO-1-Luc cells), were generated for use in high-throughput screening. Human embryonic kidney 293 (HEK293) cells (ATCC, Manassas, VA, USA) were co-transfected with a pcDNA3.1 (+) vector which confers zeocin resistance and one of two modified pGL3 luciferase reporter vectors. Construction of these vectors has been previously described [12,13,14]. To generate 4.5 kb + 220 bp HO-1-Luc cells, transfection was performed with a construct containing the −4.5 kb minimal proximal promoter fragment and 220 bp segment of the +12.5 kb enhancer of human HO-1 (pHOGL3/4.5 + 220). To generate Mut4.5kb HO-1-Luc cells, a construct containing three activity-abolishing mutations within the −4.5 kb promoter was used (Figure S1). After selection by zeocin, single cells were identified by forward and side scatter and sorted one cell per well into in a 96-well plate containing Dulbecco’s modified Eagle’s medium (Mediatech, Manassas, VA, USA) supplemented with 10% fetal bovine serum (FBS) and 1× antibiotic/antimycotic (Thermofisher, Waltham, MA, USA). All cells were grown at 37 °C in 95% humidity and 5% CO_2_. Clones arising from single cells were maintained in zeocin and screened for luciferase expression in the presence or absence of hemin. Hemin and small molecules were both prepared in the vehicle, DMSO. Prior to treatment with hemin or small molecules, cells were serum-starved in medium containing 1% FBS for 6 h. 

### 2.2. High-Throughput Luciferase Assay 

A primary high-throughput screen was developed using stable 4.5 kb + 220 bp HO-1-Luc cells seeded in 384-well plates in reduced serum media. Each well received either 10 μg/mL or 20 μM compound from a library containing small molecules generated and kindly gifted by Southern Research or with FDA-approved compounds. Luciferase induction was measured using the Bright-Glo assay system (Promega, Madison, WI, USA). A secondary counter screen utilizing Mut4.5kb HO-1-Luc cells was also developed. Positive hits identified in the primary screen were subjected to the counter screen, and compounds inducing nonspecific activation of the reporter were excluded from further testing. Hit compounds from the high-throughput screen were validated by correlating two independent experiments.

### 2.3. Western Blotting 

Kidneys and cell cultures were lysed in lysis buffer (50 mM Tris-HCl, 1 mM EDTA, 150 mM NaCl, 1% NP-40, and 0.25% sodium deoxycholate) with protease (MilliporeSigma, Burlington, MA, USA) and phosphatase (Bimake, Houston, TX, USA) inhibitors. Total protein was quantified using a bicinchoninic acid protein assay (Thermo Fisher, Waltham, MA, USA). Protein was loaded onto 10% or 12% SDS-PAGE gels, resolved, and transferred onto a PVDF membrane (MilliporeSigma, Burlington, MA, USA). Membranes were blocked in 5% milk for 1 h, and then incubated overnight with anti-cleaved caspase-3 (1:1000; #9661, Cell Signaling Technology, Danvers, MA, USA) or anti-HO-1 (1:5000; #ADI-SPA894, Enzo Life Sciences, Farmingdale, NY, USA), followed by incubation with peroxidase-conjugated anti-rabbit antibody (Kindle BioSciences, Greenwich, CT or Jackson Immunoresearch, West Grove, PA, USA). Horseradish peroxidase activity was detected using either the enhanced chemiluminescence KwikQuant detection system (Kindle BioSciences, Greenwich, CT, USA) or Konica Minolta SRX-101A film processor. Membranes were stripped and reprobed with anti-GAPDH (1:10,000; #MAB374, MilliporeSigma, Burlington, MA, USA) to confirm loading and transfer. For some blots, proteins were transferred to PVDF-FL (MilliporeSigma, Burlington, MA, USA), probed with secondary antibodies conjugated to Alexa Fluor 680 or 790 (Jackson Immunoresearch, West Grove, PA, USA), and imaged using the Licor Odyssey Imager (LI-COR, Lincoln, NE, USA). Densitometry analysis was performed using ImageStudioLite. Protein expression was normalized to GAPDH, and data are presented as fold changes relative to indicated controls.

### 2.4. In-Cell Western Assay

The HEK293 cell line was obtained from ATCC (Manassas, VA, #1573) and maintained in Dulbecco’s modified Eagle’s medium (DMEM) (Corning, Glendale, AZ, #10-013-CV) supplemented with 10% fetal bovine serum (FBS), 100 U/mL penicillin, and 100 mg/mL streptomycin in a humidified incubator with a 5% CO_2_ atmosphere at 37 °C. DMEM containing 1% FBS was used in the assay. HEK293 cells were seeded into PDL (poly-D-lysine)-coated 384-well plates (Corning #3845) at 5000 cells/well in a 20 μL volume and incubated for 2 h at 37 °C in 5% CO_2_ with high humidity for cells to settle down. Compounds were added directly to the cells using a Labcyte Echo 550 acoustic dispensing system (Labcyte), and the plates were returned to the incubator (37 °C, 5% CO_2_, high humidity). The medium was removed after a 16 h incubation period, and the cells were fixed/permeabilized with ice-cold methanol for 10 min at −20 °C. The cells were then washed once with PBS and incubated with HO-1 antibody (Enzo Life Science, Farmingdale, NY, #ADI-SPA-894) overnight at 4 °C after blocking for 30 min at room temperature with blocking buffer (PBS/10% Goat Serum/1% BSA). Two columns were set up without an HO-1 antibody to register “background”. After being washed with PBS, the cells were incubated with Alexa Fluor 488 conjugated secondary antibody for 1 h at room temperature. Finally, Hoechst staining was conducted, and the plates incubated at 4 °C overnight before reading on a Mirrorball imaging cytometer (TTP Labtech, Concord, CA, USA). The “activation (%)” was calculated by subtracting the background from HO-1 followed by normalization to Hoechst; then, 100% activity was set to Hemin E_max_. 

### 2.5. RNA Isolation

Total RNA was isolated from cells using TRIzol (Thermofisher, Waltham, MA, USA) for real-time PCR and bulk mRNA sequencing according to the manufacturer’s instructions.

### 2.6. Reverse Transcription and Real-Time PCR

Gene expression analysis was measured as previously described [15]. cDNA was generated from total RNA using the Quantitect Reverse Transcription Kit (QIAGEN, Germantown, MD, USA), and quantitative PCR was performed on a StepOnePlus Real-Time PCR System (Thermofisher, Waltham, MA, USA) to detect genes of interest using PowerUP SYBR Green Mastermix (Thermofisher, Waltham, MA, USA). The following primers were used: *NFE2L2* fwd 5′–AGTGGATCTGCCAACTACTC–3′, *NFE2L2* rev 5′– CATCTACAAACGGGAATGTCTG–3′; *HMOX1* fwd 5′–CATGACACCAAGGACCAGAG–3′, *HMOX1* rev 5′–AGTGTAAGGACCCATCGGAG–3′; *GAPDH* fwd 5′– GCCAAAAGGGTCATCATCTC–3′, *GAPDH* rev 5′–GGCCATCCACAGTCTTCT–3′. GenBank accession numbers of the top primer-BLAST products returned with the given primers are as follows: *NFE2L2* NM_006164; *HMOX1* NM_002133; *GAPDH* NM_001357943. Reactions were performed in triplicate, and relative mRNA expression was quantified using the comparative threshold cycle method. Melt curve analysis was performed at the end of each real-time PCR assay to confirm product specificity.

### 2.7. Bulk RNA Sequencing and Analysis

Single-end bulk RNA sequencing was generated by the Heflin Genomics Core (University of Alabama at Birmingham) using the Illumina platform on NextSeq500 (Illumina) with two biological replicates. Sequence alignment was conducted using STAR, and differential expression analysis was conducted using the R package DESeq2 [16], as described previously [17]. Significance was defined as an adjusted *p*-value < 0.05 and fold change >2 or <−0.2. Normalized DESeq2 counts were used for downstream analysis. Enrichment analysis for consensus target genes for transcription factors present in ENCODE [18,19] and ChEA [20] was performed with web-based Enricher [21,22,23]. Sequencing data are available within the Gene Expression Omnibus repository (GSE211130).

### 2.8. siRNA Knockdown

HEK293 cells were transfected with 50 nM SMARTpool siRNA designed to target NRF2 (Dharmacon) or a nontargeted control using RNAiMAX (Invitrogen). NRF2 knockdown was confirmed by quantitative reverse transcription PCR. 

### 2.9. Cisplatin-Induced Apoptosis Assays

HEK293 cells were pretreated with vehicle or small molecules for 8 h prior to cisplatin (50 μM) exposure. Following 24 h of cisplatin treatment, induction of HO-1 and apoptosis (via cleaved caspase-3) were evaluated by Western blot.

### 2.10. MTT Assay

Reduction of MTT following incubation with vehicle or small molecules for 24 h was assessed by the MTT assay as described previously [24]. Briefly, cells were incubated with 0.5 mg/mL MTT, which was aspirated after 1 h and replaced with dimethyl sulfoxide to solubilize the precipitated formazan. Reduction of MTT is expressed as the relative absorbance at 570 nm compared to the vehicle-treated control group.

### 2.11. In Vivo Experiments

≥10 week old C57BL/6 mice were injected intraperitoneally with 60 mg/kg of small molecule. SRI-37618 was either formulated as a free base and prepared in a saline solution containing 5% DMSO and 5% solutol or formulated as a sodium salt and prepared in saline. Formulation as a sodium salt improved solubility in saline without influencing HO-1 induction (data not shown). 6 h post administration, mice were anesthetized with isoflurane, and kidneys were harvested following cardiac perfusion with saline. All animal experiments conducted were approved by the Institutional Animal Care and Use Committee at the University of Alabama at Birmingham (Animal Project Number: IACUC-20380).

### 2.12. Immunohistochemistry

Following standard fixation in 10% neutral buffered formalin and 70% ethanol, kidneys were paraffin-embedded, and 5 μm sections were prepared. As previously described [25], sections were deparaffinized with xylene and rehydrated, followed by 30 min antigen retrieval in Trilogy (Cell Marque, Rocklin, CA, USA) at 95 °C. Endogenous peroxidase activity was blocked with 3% hydrogen peroxide solution in PBS for 10 min at room temperature. Blocking was performed with a solution of 0.2% nonfat dry milk, 1% BSA, and 0.3% Triton X-100 in PBS for 1 h at room temperature. HO-1 staining was performed overnight at 4 °C (1:250–1:500; Enzo Life Sciences, Farmingdale, NY, USA). Following washing with PBS/Tween-20 (PBST), secondary antibody (1:500; Jackson ImmunoResearch Laboratories, West Grove, PA, USA) staining was performed for 1 h at room temperature. Sections were then developed using chromogen substrate (Vector Laboratories, Burlingame, CA, USA) according to the manufacturer’s instructions, washed with water, dehydrated, and mounted (Xylene Mounting Media; Protocol, Kalamazoon, MI, USA). All images were acquired using the Keyence BZ-X710 (Keyence Corporation, Itasca, IL, USA).

### 2.13. Statistical Analysis

All experiments were performed in independent triplicates unless otherwise noted. Results are expressed as the mean + SEM. One- or two-way ANOVA and follow-up tests for multiple comparisons were employed for analysis between three or more groups (*p* < 0.05 was considered statistically significant).

## 3. Results

### 3.1. High-Throughput Screen and Lead Generation 

**Figure 1:**
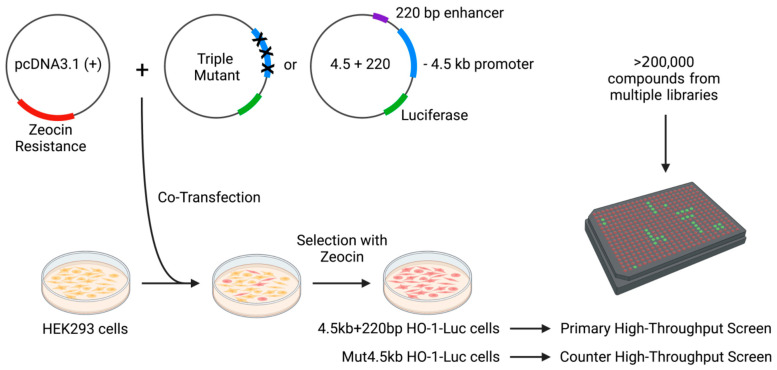
Schematic for the creation of 4.5 kb + 220 bp HO-1-Luc and Mut4.5kb HO-1-Luc stable cell lines and corresponding high-throughput screens. Human embryonic kidney 293 (HEK293) cells were stably co-transfected with pcDNA3.1 (+) which conferred zeocin resistance and modified pGL3 luciferase reporter vector. A pHOGL3/4.5 + 220 wildtype (labeled as 4.5 + 220) construct containing the −4.5 kb minimal proximal promoter fragment and 220 bp segment of the +12.5 kb enhancer of human HO-1 gene was used in the creation of 4.5 kb + 220 bp HO-1-Luc cells. A pHOGL3/4.5 triple-mutant construct containing three promoter activity abolishing mutations (labeled Triple Mutant) was used to generate Mut4.5kb HO-1-Luc cells. Transfected cells surviving zeocin selection were plated in a 96- or 384-well format to facilitate high-throughput screening of compound libraries for luciferase activity, indicating HO-1 induction (primary screen). Compounds producing luciferase activity in the counter screen with Mut4.5kb HO-1-Luc cells were considered false positives and eliminated from further investigation.

**Figure 2:**
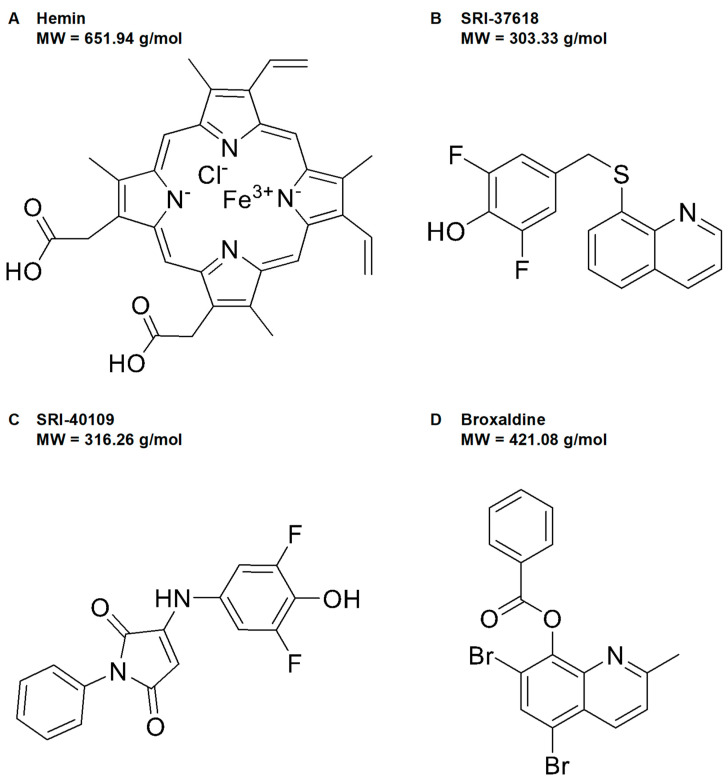
Structures of (**A**) hemin and (**B**–**D**) three small molecules identified by high−throughput screening for their ability to induce luciferase activity driven by the human HO-1 gene. MW = molecular weight.

A high-throughput screen was performed on a library of 200,441 total compounds for the ability to induce HO-1 in a luciferase reporter system (Figure 1). The libraries were compiled by Southern Research and included ~4000 FDA-approved molecules with the potential for repurposing as well as other commercially available and novel molecules synthesized by Southern Research. The primary high-throughput screen, utilizing 4.5 kb + 220 bp HO-1-Luc cells, was used for the initial screening of hit compounds. Compounds activating the reporter construct to levels of ≥70% max activation of 5 µM hemin (Figure 2A), the canonical HO-1 inducer [26], were considered active. Initial positive hits were assayed in a counter high-throughput screen utilizing Mut4.5kb HO-1-Luc cells to eliminate false positives that activate HO-1 through nonspecific response elements. 

**Figure 3:**
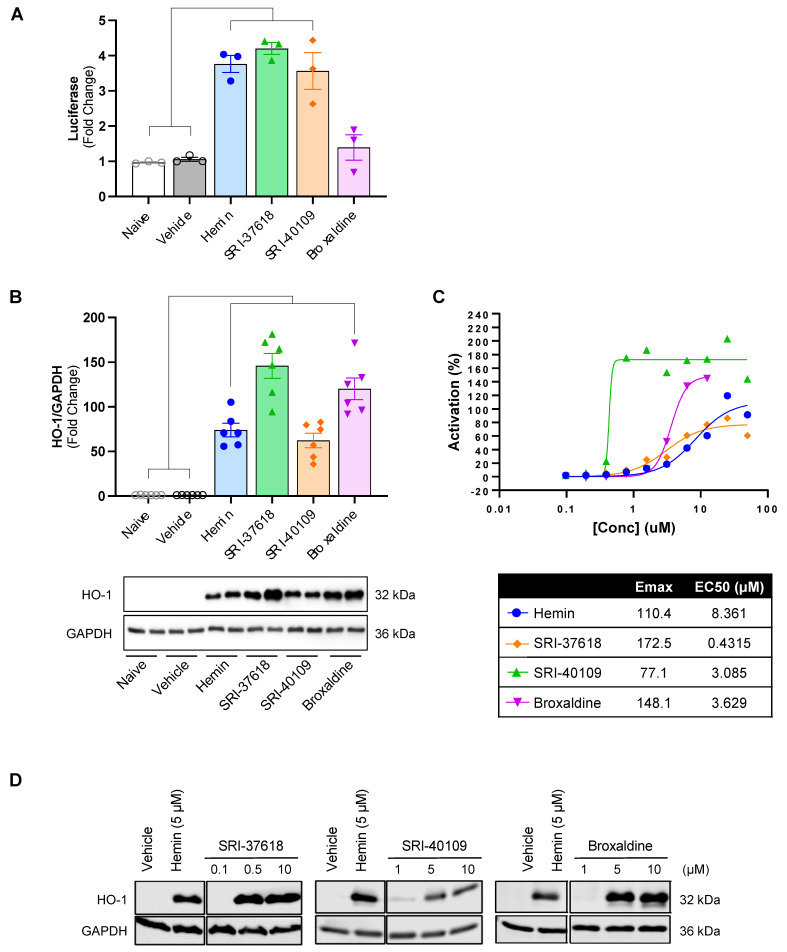
(**A**) Luciferase activity induced in 4.5 kb + 220 bp HO-1-Luc cells by small molecules normalized to baseline luciferase signal in naïve, untreated cells. DMSO and hemin are included as vehicle and positive controls. (**B**) Densitometry of Western blot of 4.5 kb + 220 bp HO-1-Luc cells for HO-1 and loading control GAPDH under the same experimental conditions as luciferase assay. HO-1/GAPDH is expressed as fold change compared to vehicle. Lower panel displays a representative Western blot. (**C**) In-cell Western and (**D**) standard Western blot for endogenous HO-1 induction in HEK293 cells exposed to increasing concentrations of compound. Maximum HO-1 activation (E_max_) and effective concentration to achieve 50% activation (EC_50_) are listed in lower panel of (**C**). Data analyzed by one-way ANOVA followed by Dunnett’s test for multiple comparisons.

In this process, two hit compounds, SRI-36825 and SRI-37619, induced HO-1 and exhibited low toxicity in vitro. SRI-36825 was derivatized into a new lead compound, SRI-37618, and SRI-37619 led to SRI-40109, two novel small molecule candidates representing two distinct chemical scaffolds (Figure 2B,C). High-throughput screening also identified FDA-approved broxaldine as a candidate small-molecule inducer of HO-1 (Figure 2D). The luciferase assay in the stable cell lines was repeated with these hit compounds to confirm induction of HO-1 through the human promoter and enhancer elements (Figure 3A). Consistent with the high-throughput screen data, induction of luciferase activity was not observed in Mut4.5kb HO-1-Luc cells (Figure S2). Relative to hemin, SRI-37618 and SRI-40109 induced similar luciferase activity in the primary screen. Although broxaldine did not induce statistically significant luciferase expression compared to controls, repurposing an FDA-approved drug would save considerable cost and effort in transitioning to clinical testing; hence, we did not use the luciferase assay as the sole criterion for further screening. Additional cells were plated in parallel and treated to confirm HO-1 protein expression in the stable cells (Figure 3B). Broxaldine induced higher HO-1 protein than hemin but lower luciferase activity (Figure 3A,B).

### 3.2. Induction of Endogenous HO-1 by SRI-37618 and SRI-40109 Does Not Depend on Oxidant Stress

**Figure 4:**
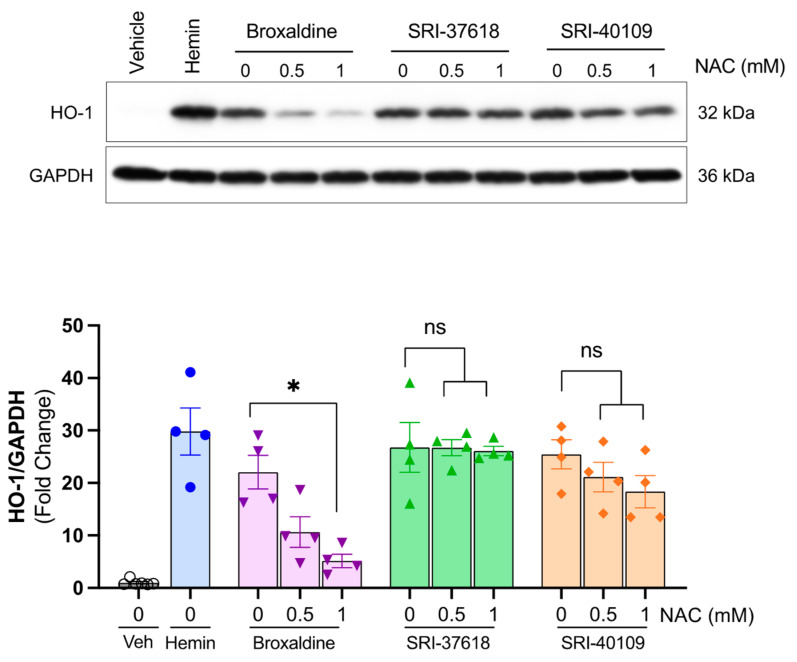
Top panel: Representative Western blot analysis for HO-1 in HEK293 cells treated with broxaldine (5 µM), SRI-37618 (0.5 µM), SRI-40109 (5 µM), hemin (5 µM), or vehicle (DMSO) in the presence of 0, 0.5, or 1 mM N-acetylcysteine (NAC). Lower panel: HO-1 induction normalized to corresponding GAPDH expression and expressed in arbitrary units relative to vehicle control. Each dot represents an independent experiment (n = 4). Data analyzed with one-way ANOVA and Šídák’s multiple-comparison test. Error bars display SEM. ns *=* not significant, * *p* < 0.05.

To validate the endogenous activity of candidate small molecules, HEK293 cells were treated with increasing concentrations of each small molecule or hemin, a positive control (Figure 3C). Endogenous HO-1 is robustly induced in a dose-dependent manner by SRI-37618, SRI-40109, and broxaldine as determined by fluorescence-based in-cell Western blotting (Figure 3C), which was validated using traditional Western blotting (Figure 3D). To establish whether the HO-1 induction resulting after treatment with candidate small molecules was secondary to oxidant stress, in vitro assays were performed in the presence of a general antioxidant N-acetylcysteine (NAC) (Figure 4). In the presence of up to 1 mM NAC, HO-1 protein expression was preserved in HEK293 cells treated with SRI-37618 and SRI-40109, but diminished in the case of broxaldine. 

### 3.3. Genes Upregulated by Hemin, SRI-37618, SRI-40109, and Broxaldine Share Common NRF2-Binding Motifs

**Figure 5:**
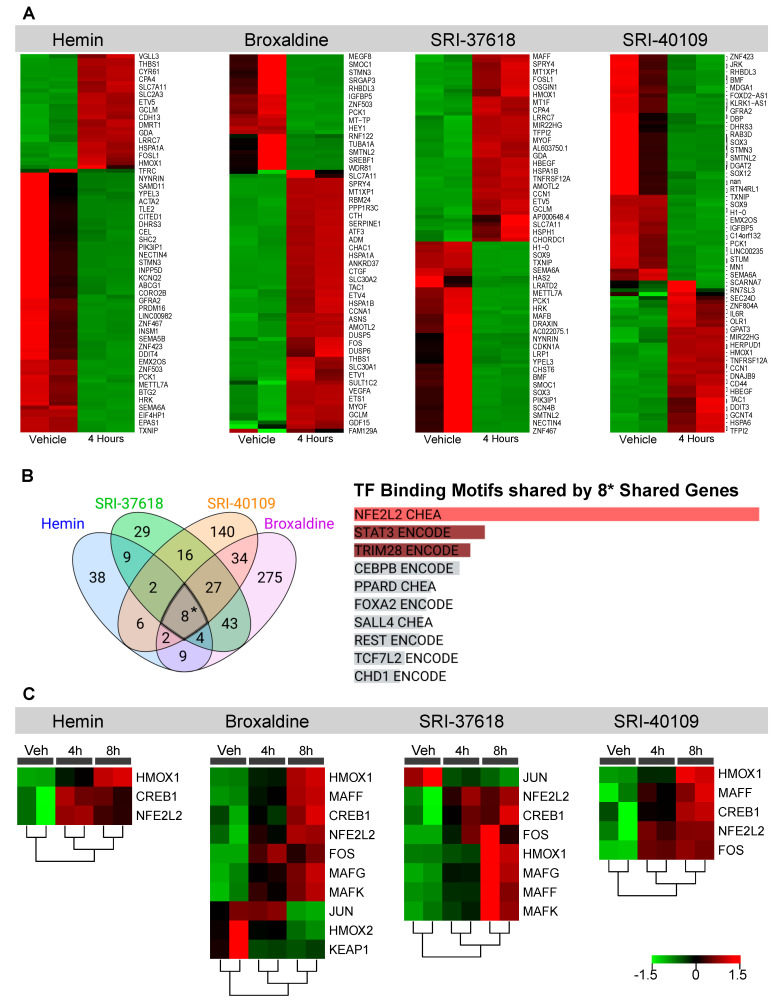
(**A**) Heatmap of the top 100 differentially expressed genes in HEK293 cells exposed to hemin, broxaldine, SRI−37618, or SRI−40109 for 4 h. Red coloration depicts genes upregulated, and green depicts genes downregulated relative to vehicle control. (**B**) Left panel: Venn diagram displaying the number of shared genes upregulated at 4 h by all four treatment conditions relative to vehicle control. * Eight shared genes upregulated at 4 h: *CPNE9*, *GCLM*, *GCNT4*, *HMOX1*, *SLC7A11*, *SERPINE1*, *OSGIN1*, and *NMRAL2P*. Right panel: Enrichr analysis for eight shared genes showing top 10 consensus transcription factor-binding sites from ENCODE and ChEA ChIP-X. Bar graph is ranked by *p*-value. (**C**) Heatmap of the top NRF2-related genes from the list of significant (*p* < 0.05) genes from DeSeq2 analysis of each compound compared to vehicle control. Gene targets mapped include *CREB1*, *CRYZ*, *FOS*, *FXYD2*, *GSTA2*, *HMOX1*, *HMOX2*, *JUN*, *KEAP1*, *MAFF*, *MAFG*, *MAFK*, *NFE2L2*, *POR*, *PRKCA*, *PRKCB*, and *UGT1A6.* Red and green coloration depicts up- and downregulation, respectively. HMOX1, the gene for HO-1, is highlighted in yellow in heatmaps.

**Figure 6:**
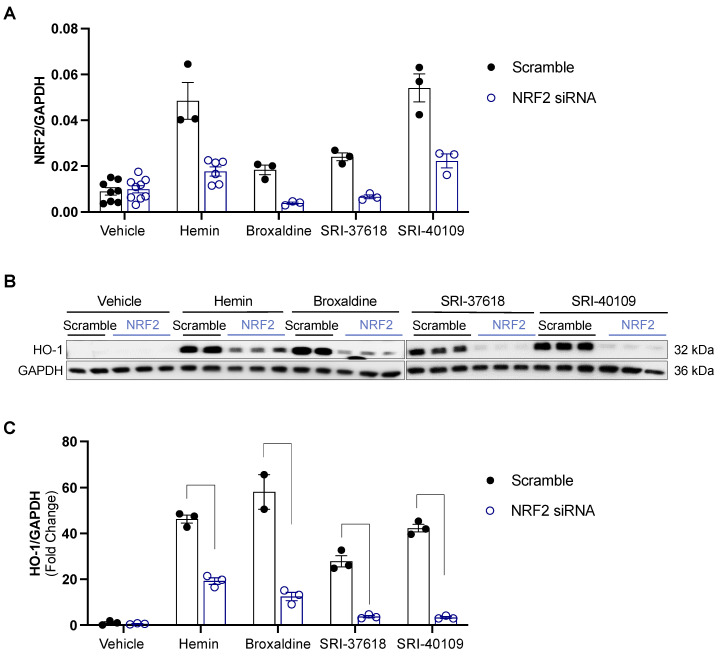
(**A**) Confirmation of NRF2 knockdown by real-time PCR. NRF2 gene expression is normalized to housekeeping gene GAPDH. (**B**) Western blot for HO-1 in cells exposed to compounds in the presence of NRF2-targeted siRNA or scramble siRNA control. (**C**) HO-1 protein expression normalized to corresponding GAPDH control expressed relative to scramble siRNA-treated vehicle control. Data analyzed by two-way ANOVA with Šídák’s multiple-comparison test.

Bulk RNA sequencing was performed on HEK293 cells exposed to vehicle control, hemin, SRI-37618, SRI-40109, or broxaldine for 4 or 8 h. Compared to the vehicle-treated control group, all treatment groups exhibited significantly increased HO-1 gene expression with a fold change of >2 and adjusted *p*-value of <0.05, corroborating increases in HO-1 promoter activation and protein level. In addition to upregulation of *HMOX1*, the greater transcriptional profiles of cells were influenced by treatment with each candidate molecule (Figure 5A and Figure S3). Although each compound elicited a unique transcription profile, increased expression of several genes was shared among all treatment groups (Figure 5B). Transcripts for *HMOX1*, *NMRAL2P*, *OSGIN1*, *SLC7A11*, *GCLM*, *CPNE9*, *SERPINE1*, and *GCNT4* were upregulated by all candidate molecules and hemin after 4 h compared to vehicle control (Figure 5B). Enrichment analysis of these transcripts showed significant (adjusted *p*: 5.09 × 10^−4^) association with consensus binding sites for NRF2 defined by the ChEA and ENCODE datasets of ChIP-seq data. This association is driven primarily by *HMOX1*, *SERPINE1*, *OSGIN1*, *SLC7A11*, and *GCLM* (Figure 5B and Figure S4, Table S1). NRF2 is a well-described transcription factor that is known to regulate the expression of several antioxidant genes, including *HMOX1* [27]. We also looked for enrichment of NRF2 pathway-associated genes (*CREB1*, *CRYZ*, *FOS*, *FXYD2*, *GSTA2*, *HMOX1*, *HMOX2*, *JUN*, *KEAP1*, *MAFF*, *MAFG*, *MAFK*, *NFE2L2*, *POR*, *PRKCA*, *PRKCB*, and *UGT1A6*) amongst the significant (*p* < 0.05) differentially expressed genes and found significant upregulation at the 4 and 8 h timepoints (Figure 5C). We further confirmed the role of NRF2 in small-molecule induction of HO-1 by knocking down *NFE2L2* via siRNA prior to small-molecule exposure. Knockdown of *NFE2L2* in transfected HEK293 cells was confirmed by real-time PCR 8 h following transfection (Figure 6A). Following transfection, cells were treated with vehicle, hemin, or small molecules, and HO-1 expression was analyzed by Western blot 16 h post treatment (Figure 6B). Transfection with *NFE2L2* siRNA mitigated HO-1 induction by hemin and all small molecules tested, with the greatest effect on SRI-37618 and SRI-40109 (Figure 6C).

### 3.4. SRI-37618, SRI-40109, and Broxaldine Pretreatment Confer Protection against Cisplatin-Induced Apoptosis In Vitro

**Figure 7:**
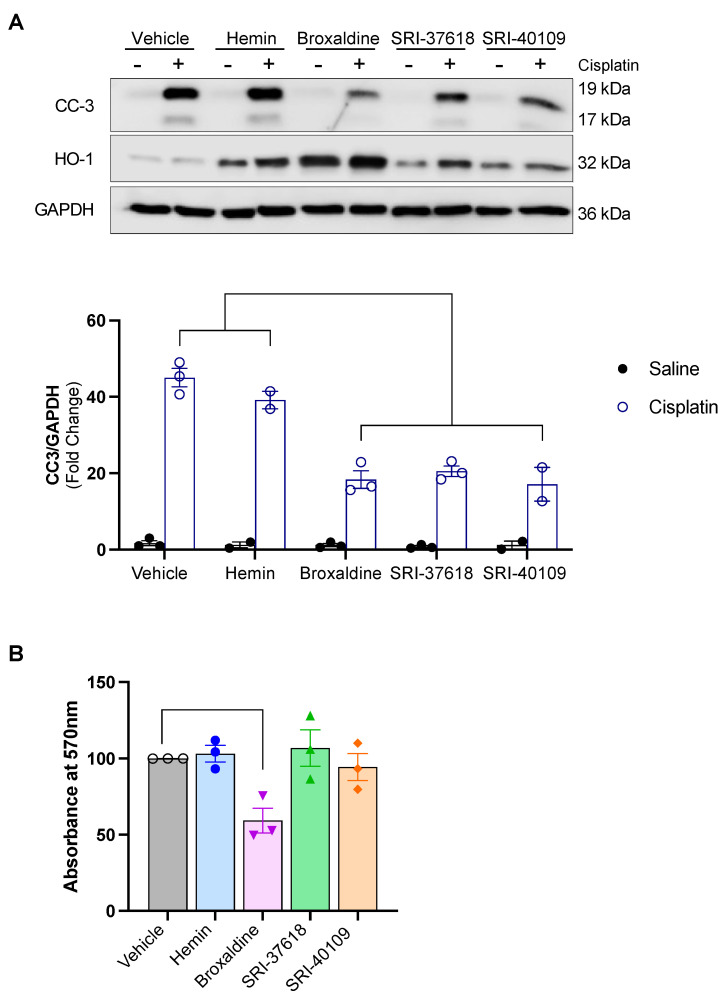
(**A**) Western blot for cleaved caspase-3 (CC-3) and heme oxygenase-1 (HO-1) in HEK293 cells pretreated with listed compounds or controls for 8 h prior to cisplatin exposure. (**B**) MTT assay of HEK293 cells treated with compounds or vehicle control. Absorbance at 570 nm relative to vehicle control reflects the conversion of MTT (3-(4, 5-dimethylthiazolyl-2)-2, 5-diphenyltetrazolium bromide) into formazan and is used as an indirect proxy for relative cell viability. Data analyzed by one-way ANOVA followed by Dunnett’s test for multiple comparisons.

We next assessed the ability of small molecule-induced HO-1 to protect against cisplatin-induced cytotoxicity. Our laboratory and others have described the protective effect of HO-1 in cisplatin-mediated nephrotoxicity in preclinical models [4,28,29]. Pretreatment with 5 mM hemin was used as a positive control for HO-1 induction in this assay. Although hemin is a potent inducer of HO-1, heme-containing compounds can cause acute kidney injury through damage of multiple intracellular targets [30]. As expected, subsequent exposure to cisplatin and release of additional heme from dying cells resulted in significant expression of apoptotic marker cleaved caspase-3 (CC-3), highlighting the need for therapeutic inducers of endogenous HO-1 that do not rely on heme itself (Figure 7A). Following induction of endogenous HO-1 through pretreatment with broxaldine, SRI-37618, and SRI-40109, cisplatin-induced apoptosis was significantly blunted compared to saline controls (Figure 7A). As a correlate to protection against apoptosis, we also examined the effects of the small molecules on cell viability using an MTT assay (Figure 7B). Notably, broxaldine itself diminished the ability of cells to reduce the tetrazolium dye to formazan at the given dose measured by the MTT assay. However, broxaldine pretreatment alone did not result in significant CC-3 induction, suggesting that the aberrant MTT assay result is not attributable to apoptosis. Considering that the MTT assay employed to assess cell viability depends on mitochondrial respiration, this result may also be indicative of changes in mitochondrial metabolism induced by broxaldine rather than true cytotoxicity. Nevertheless, such influences are important when designing or repurposing drugs. 

### 3.5. In Vivo Induction of Endogenous HO-1 in the Kidney 

**Figure 8:**
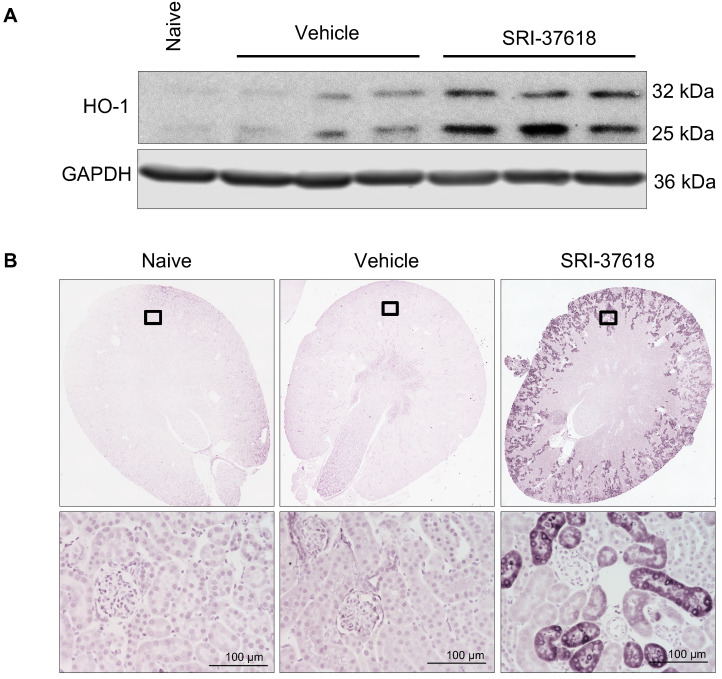
In vivo delivery and induction of HO-1 in the kidney assessed after 6 h of small-molecule delivery. (**A**) Western blot for HO-1 in whole kidney lysates of mice injected intraperitoneally with 60 mg/kg of small molecule SRI-37618 formulated as a free base in a vehicle solution of 5% DMSO, 5% solutol in saline. Membranes were stripped and probed for GAPDH. Each lane represents an individual mouse (n = 3) (**B**) Representative immunohistochemistry for HO-1 in kidney sections of mice 6 h after intraperitoneal injection with 60 mg/kg SRI-37618 prepared as a salt solution capable of being dissolved and delivered in saline vehicle. Top panel showcases entire kidney section (20×); the cortical insets are displayed in the lower panel. Scale bar = 100 µm.

Given the promising in vitro studies with the candidate small molecules, we next investigated the in vivo induction of HO-1 by the small molecules. We first chose SRI-37618 as the lead compound for evaluation because of its potent ability to induce endogenous HO-1 expression (Figure 3B–D) and target the human *HMOX1* promoter and enhancer constructs (Figure 3A). When administered intraperitoneally at a dose of 60 mg/kg, SRI-37618 resulted in HO-1 protein induction in the kidney within 6 h, as revealed by Western blot analysis of whole-kidney lysates and immunohistochemistry of kidney sections (Figure 8A,B). HO-1 induction appeared to be particularly robust in the cortical proximal tubules. No grossly observable signs of acute distress or changes in behavior were seen in mice during the treatment period (data not shown). 

## 4. Discussion

Heme oxygenase-1, encoded by the *HMOX1* gene, is ubiquitous and required for survival. In both animal models and humans, HO-1 is strongly induced in the setting of AKI [3,31], with plasma and urine HO-1 being considered a biomarker for AKI [32]. Polymorphisms in the human *HMOX1* promoter correlate with HO-1 expression and outcomes in AKI [33,34,35]. One particularly well-documented and relevant genetic association is the increased risk of developing AKI among white patients undergoing cardiopulmonary bypass surgery with polymorphisms resulting in a longer GT repeat region of their *HMOX1* gene promoter [35]. Genetic or pharmacologic manipulation of the HO-1 system in animal models impacts AKI progression and recovery. As reviewed by Nath et al. [3], HO-1 deficiency or inhibition worsens renal structure and function, and increased expression is protective in models of AKI. In fact, many interventions that have been tested in preclinical models of AKI (e.g., α-MSH, EPO, IL-10, and NGAL) are potent inducers of HO-1 and mediate their effects, at least in part, through HO-1 induction [36,37,38,39,40]. The protective effects of HO-1 induction are largely attributed to the degradation of highly reactive, oxidized heme into vasodilator CO and biliverdin, which is subsequently converted to free-radical scavenger bilirubin. The reaction simultaneously liberates iron which induces ferritin, another cytoprotective mediator in AKI [25]. Additionally, CO can act as an immunomodulator, enhancing anti-inflammatory IL-10 and blocking proinflammatory TNF-α [41,42]. The pleiotropic effects of the products of HO-1-catalyzed heme degradation have made the HO-1 system a favorable target for intervention in the pathogenesis of AKI. The innovative approach of inducing HO-1 itself is particularly attractive because it enables the simultaneous exploitation of heme degradation and the subsequent cytoprotective properties of the reaction products.

Pharmacologic manipulation of the HO-1 system through delivery of heme analogs or products has proven to be a challenging approach for producing clinical benefit. For example, heme arginate, while approved for use in acute intermittent porphyria, can cause many off-target effects and unwanted adverse events, including AKI [43]. Low concentrations of CO are protective in preclinical models, but the toxic effect of this gas on mitochondria is a major limitation in clinical application [44]. Direct pharmacologic induction of endogenous HO-1 using small molecules provides a possible solution to overcoming these limitations and has broad applicability for hospitalized patients who are at risk for developing AKI. 

To identify small-molecule inducers of HO-1 that are not substrate analogs and that result in the production of all the antioxidant products of heme degradation, we developed a high-throughput cell-based assay to screen a large library of compounds for their ability to induce HO-1 expression. Importantly, high-throughput screens utilized cell lines expressing luciferase reporter constructs under the control of the promoter and enhancer of the human HO-1 gene. While human and mice HO-1 transcription factors share greater than 90% homology, consideration of interspecies differences is critical for improving the feasibility of translating preclinical findings to patients (as reviewed by Sikorski et al. [45]). 

We identified three hit compounds, SRI-37618, SRI-40109, and broxaldine, that induced expression of human HO-1. Given that these compounds are from three chemical classes, their exact drug profiles likely differ. Nevertheless, SRI-37618 and SRI-40109 were capable of activating the −4.5 kb minimal proximal promoter and 220 bp segment of the +12.5 kb enhancer of human *HMOX1*, and all three induced endogenous HO-1 protein. In the case of SRI-37618 and SRI-40109, this induction occurred even in the presence of antioxidant, NAC, suggesting that the effects seen were not merely a nonspecific induction secondary to the production of reactive oxygen species. The HO-1 induction by broxaldine, on the other hand, was significantly reduced in the presence of NAC. Taken with the luciferase activity of 4.5 kb + 220 bp HO-1-Luc cells and the endogenous HO-1 protein levels in HEK293 cells induced by broxaldine, these results suggest that there are likely additional mechanisms at play contributing to the levels of endogenous HO-1 protein induction observed following broxaldine delivery. 

Bulk RNA sequencing revealed that, although there was some heterogeneity between the gene expression profiles elicited by each compound at the given concentrations and timepoints, all three compounds and hemin upregulated NRF2-associated genes, and five of the eight genes significantly upregulated by all treatment conditions at 4 h showed strong enrichment of NRF2-binding sites. This list included *HMOX1*. NRF2 is a transcription factor known to promote expression of genes containing proximal antioxidant response elements (AREs), such as *HMOX1*. siRNA knockdown of *NFE2L2* in HEK293 cells dampened, but did not completely ablate the production of HO-1 by candidate compounds, supporting previous observations that, while NRF2 can significantly enhance human HO-1 gene activation indirectly through regulation of AP-1 family transcription factors [14,46], it is not a requirement, and HO-1 can also be induced through NRF2-independent pathways (as is the case with kaempferol [47] and nitrolinoleic acid [48]). It should be noted that there is debate surrounding the physical interaction of NRF2 and the *HMOX1* promoter in humans. Compounds may also be influencing upstream components of the NRF2 pathway, its translocation, or post-translational modifications of transcripts induced that are not captured by this type of analysis. The diversity of HO-1 inducers discovered to date highlights the complexity of human HO-1 regulation. As investigation of novel compounds identified through unbiased screens continues to expand therapeutic options, mechanistic studies are needed to improve our understanding of HO-1 regulation and action.

While the therapeutic value of HO-1 in kidney injury is well established, we wanted to validate that the HO-1 induced by SRI-37618, SRI-40109, and broxaldine is functional and confers a protective advantage. All three candidate compounds mitigated cisplatin-induced apoptosis in vitro, providing further evidence that the HO-1 protein induced by these small molecules is not only functional, but that these compounds could also have potential to be used to mitigate the adverse kidney affects associated with cisplatin chemotherapy, which would be a great benefit to the approximately 25% of patients receiving cisplatin treatment for solid organ cancers that develop AKI or CKD. Of course, localized delivery of the compounds to the kidney may be more appropriate than systemic delivery in these cases to minimize the likelihood that the antiapoptotic effects of the compounds would diminish the efficacy of cisplatin as a chemotherapeutic agent. Additionally, recent studies have suggested HO-1 may have noncanonical functions outside of its catalytic activity that may promote tumor growth [49]. Thorough evaluation of this potential risk against the protective effect against AKI will be needed prior to clinical administration, and may likely need to be individualized to each patient and clinical scenario.

The downstream antiapoptotic effect of small-molecule HO-1 induction likely provides clinical benefit to several other kidney pathologies as well, such as sepsis-associated AKI which is partially characterized by apoptosis of proximal and distal tubules [50,51], and accounts for nearly half of AKI incidences [52]. Of note, broxaldine negatively impacted cell viability as assessed through MTT assay, an effect that may reflect a nonapoptotic mechanism of cell death or be an incidental change in mitochondrial metabolism that influences this indirect method of measuring viability. Preliminary in vivo feasibility was evaluated with SRI-37618 as this compound exhibits greater maximum induction of HO-1 activity than hemin and is very potent with an EC_50_ of 0.4315 mM (Figure 3C). Intraperitoneal delivery of SRI-37618 successfully induced HO-1 protein expression in the kidney without producing grossly observable side-effects in the acute phase. 

## 5. Conclusions

This study not only provided a high-throughput methodology for the discovery of additional inducers of human HO-1, but also identified SRI-37618 as a promising therapeutic candidate to be optimized for clinical use as either a prophylactic or a therapeutic for AKI. Pharmacologic inducers of endogenous HO-1 also serve as a tool for better understanding the molecular mechanisms governing AKI. Future studies evaluating downstream effects of HO-1 reaction products following small-molecule delivery will provide invaluable insight into kidney pathophysiology.

## Data Availability

Sequencing data are available within the Gene Expression Omnibus repository (GSE211130). Other data presented in this study are available on request from the corresponding author.

## Supplementary Materials

The following supporting information can be downloaded at https://www.mdpi.com/article/10.3390/antiox11101888/s1: Figure S1. Luciferase reporter constructs; Figure S2. Luciferase expression in primary and secondary counter luciferase assays; Figure S3. Differentially expressed genes following 8 h of compound exposure; Figure S4. Enrichr analysis for eight shared genes upregulated by hemin, SRI-37618, SRI-40109, and broxaldine at 4 h; Table S1. Consensus target genes for transcription factors present in ENCODE and ChEA.
