# Supplementary material for: Identification of Cytoprotective Small-Molecule Inducers of Heme-Oxygenase-1
Source: Antioxidants (Basel). 2022 Sep 23;11(10):1888. doi: 10.3390/antiox11101888 (PMC9598442; doi:10.3390/antiox11101888)
Supplement: Supplementary file 1 [file antioxidants-11-01888-s001.zip › antioxidants-1900975-supplementary.pdf]

Figure S1

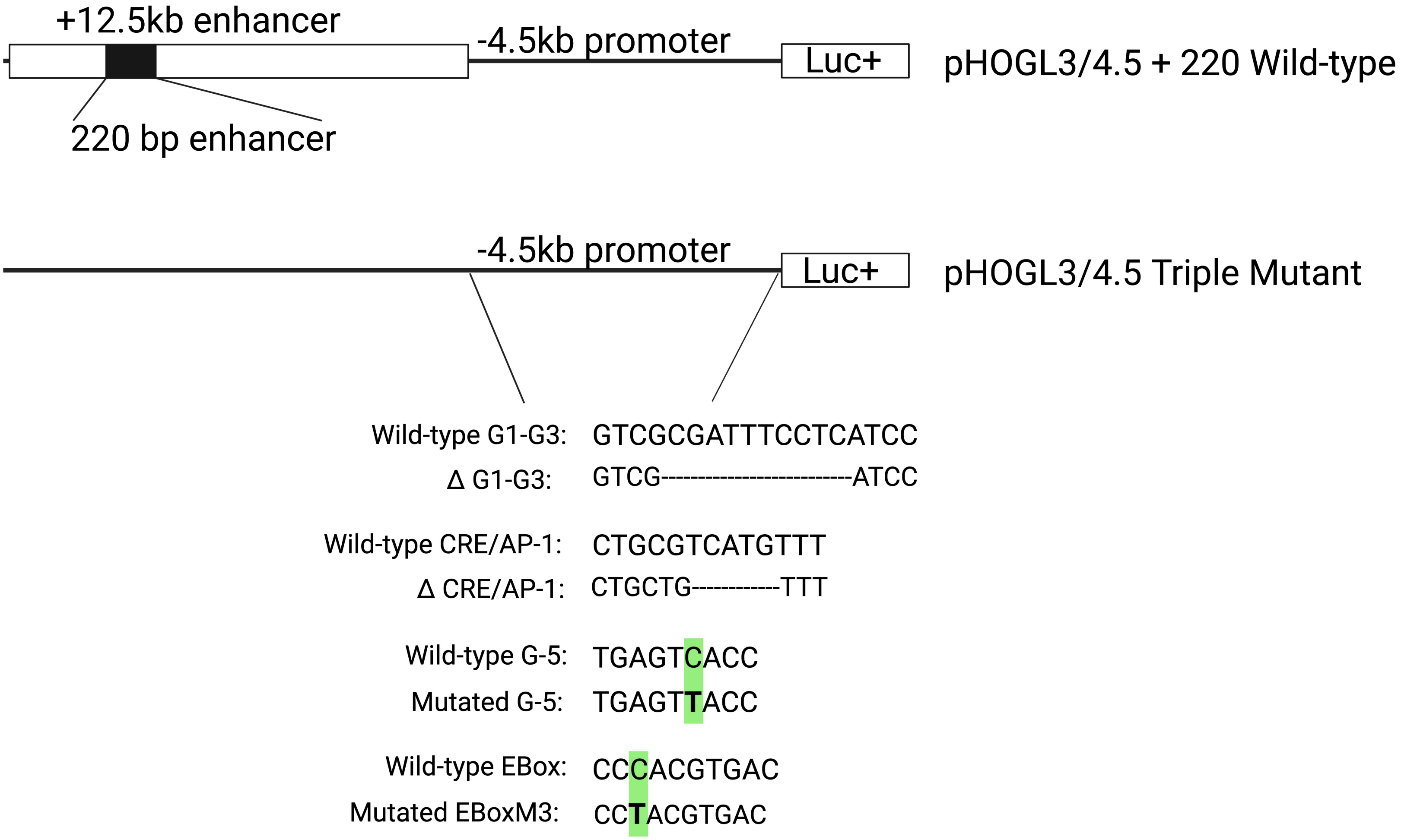

Figure S1. Reporter constructs used for the generation of 4.5kb+220bp HO-1-Luc and Mut4.5kb HO-1-Luc stable cell lines and high throughput screen for small molecules that activate the human HO-1 promoter/enhancer. The wild-type pHOGL3 plasmid has intact sequences while the triple mutant contains deletions in the cAMP-response element/AP-1 (CRE/AP-1) and the G1-3 region, and point mutations in the Ebox and G-5 as indicated by the Δ and highlighted changes respectively. The triple mutant was used to counter screen compounds to ensure specific activity through the promoter rather than targeting response elements with other promoters. Therefore, compounds that activated the native promoter and the triple mutant were considered non-specific and rejected.

Figure S2

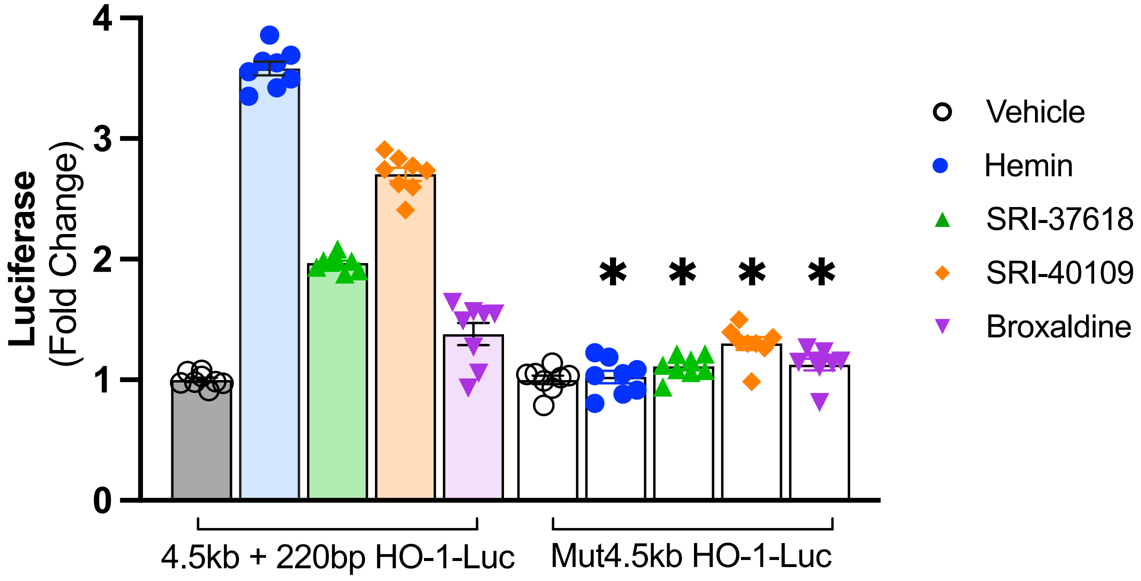

Figure S2. Luciferase expression induced in stable cell line 4.5kb + 220bp HO-1-Luc or Mut4.5kb HO-Luc cells treated with vehicle (DMSO), hemin (5 µM), SRI-37618 (0.5 µM), SRI-40109 (5 µM), or broxaldine (5 µM) for 16 hours. Luciferase expression is normalized to that seen in vehicle (DMSO)-treated control. Data analyzed by one-way ANOVA followed by Tukey's multiple comparisons test. *$p < 0.05$ indicates significance compared to expression in intact 4.5kb+220bp HO-1-Luc cells treated with corresponding compound.

Figure S3

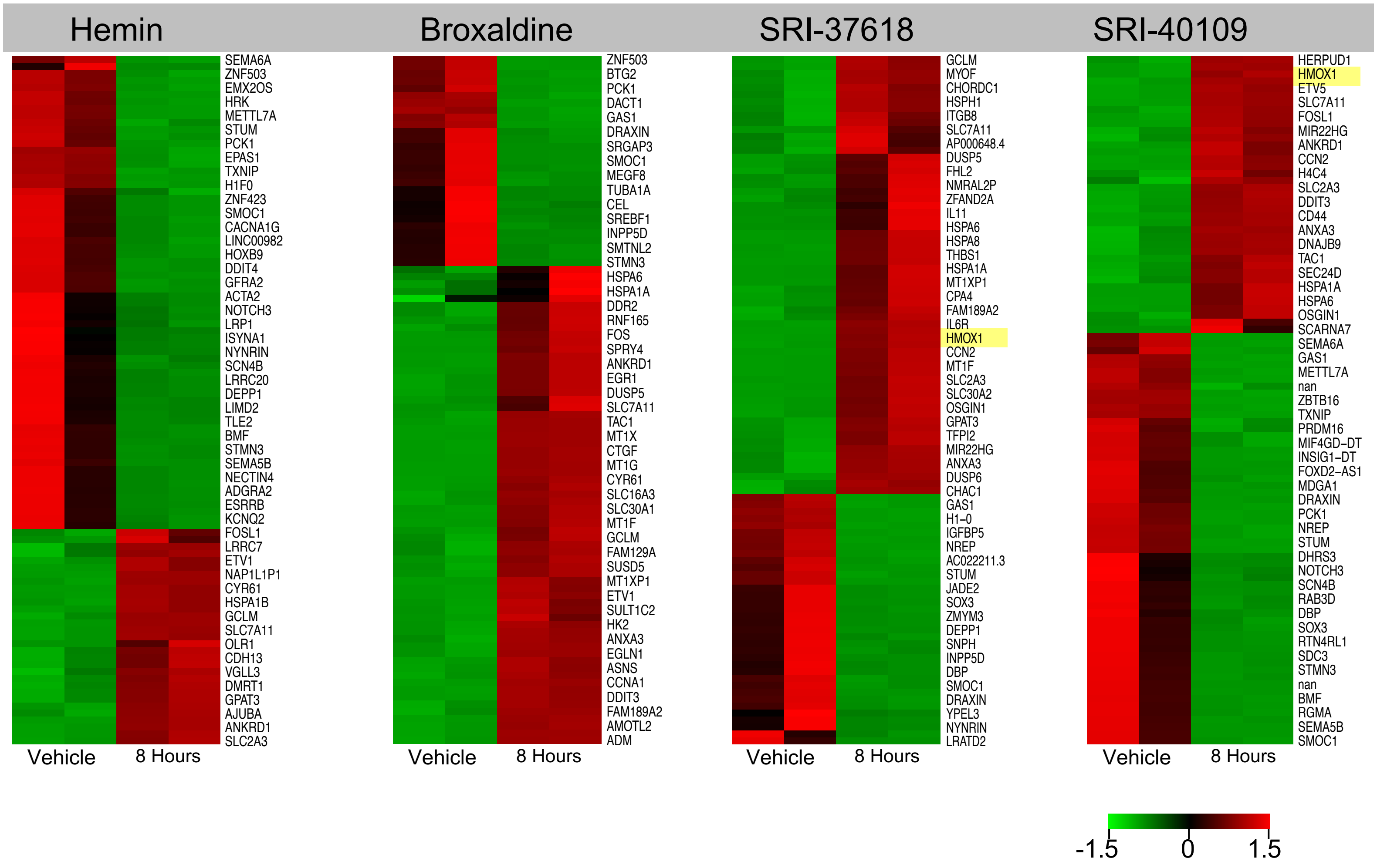

Figure S3. Heatmap of the top 100 differentially expressed genes in HEK293 cells exposed to hemin, broxaldine, SRI-37618, or SRI-40109 for 8 hours. Red coloration depicts genes that are upregulated, and green depicts genes downregulated relative to vehicle control. *HMOX1*, the gene for HO-1, is highlighted in yellow in heatmaps.

Figure S4

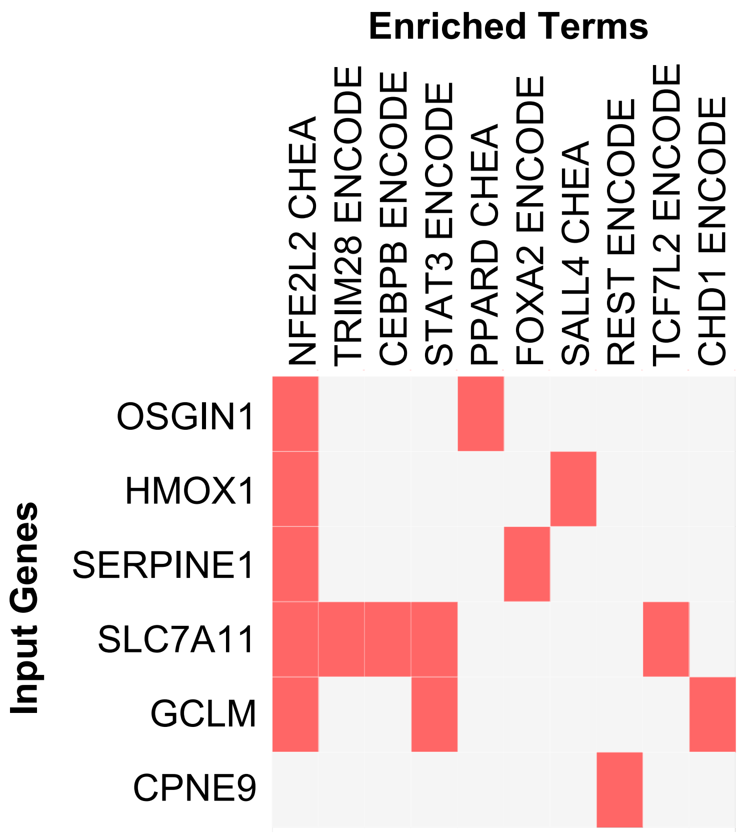

Figure S4. Enrichr analysis for 8 shared genes upregulated by hemin, SRI-37618, SRI-40109, and broxaldine at 4 hours. Input genes are listed in rows, enriched terms within ENCODE and ChEA CHIP-x are the columns, and cells in the matrix indicate if a gene is associated with a term. GCLM, GCNT4, and NMRAL2P did not return any associations within the consensus dataset.

Table S1. Enrichr analysis for 8 shared genes upregulated by hemin, SRI-37618, SRI-40109, and broxaldine at 4 hours (*CPNE9*, *GCLM*, *GCNT4*, *HMOX1*, *SLC7A11*, *SERPINE1*, *OSGIN1*, and *NMRAL2P*). Consensus target genes for transcription factors that are present in ENCODE and ChEA. The p-value is computed from Fisher exact test and adjusted p-value is computed using Benjamini-Hochberg method. To derive ranking, the Fischer exact test for many random gene sets computes a mean rank and standard deviation from the expected rank for each term in the gene-set library and calculates a z-score to assess the deviation from the expected rank. The combined score is derived by taking the log of the p-value from the Fischer exact test and multiplying that by the z-score of the deviation from expected rank.

Term	P-value	Adjusted P-value	Odds Ratio	Combined Score	Genes
NFE2L2 CHEA	1.7E-05	5.1E-04	31.10	341.55	SERPINE1;OSGIN1;HMOX1;SLC7A11;GCLM
STAT3 ENCODE	3.2E-02	4.2E-01	8.88	30.64	SLC7A11;GCLM
TRIM28 ENCODE	4.7E-02	4.2E-01	23.86	72.94	SLC7A11
CEBPB ENCODE	5.6E-02	4.2E-01	19.83	57.09	SLC7A11
PPARD CHEA	1.1E-01	5.4E-01	9.91	22.02	OSGIN1
FOXA2 ENCODE	1.2E-01	5.4E-01	8.92	18.95	SERPINE1
SALL4 CHEA	1.3E-01	5.4E-01	7.92	15.96	HMOX1
REST ENCODE	1.4E-01	5.4E-01	7.33	14.25	CPNE9
TCF7L2 ENCODE	2.1E-01	5.6E-01	4.76	7.42	SLC7A11
CHD1 ENCODE	2.3E-01	5.6E-01	4.22	6.14	GCLM
RCOR1 ENCODE	2.5E-01	5.6E-01	3.93	5.47	OSGIN1
CEBPD ENCODE	2.6E-01	5.6E-01	3.75	5.08	SLC7A11
GATA2 CHEA	2.7E-01	5.6E-01	3.56	4.66	SERPINE1
BCLAF1 ENCODE	2.9E-01	5.6E-01	3.22	3.94	GCLM
USF2 ENCODE	3.3E-01	5.6E-01	2.82	3.15	HMOX1
TCF3 CHEA	3.4E-01	5.6E-01	2.70	2.93	GCLM
SPI1 CHEA	3.5E-01	5.6E-01	2.56	2.68	OSGIN1
AR CHEA	3.6E-01	5.6E-01	2.47	2.50	SLC7A11
SIN3A ENCODE	3.7E-01	5.6E-01	2.38	2.36	SLC7A11
HNF4A ENCODE	4.1E-01	5.6E-01	2.10	1.87	SERPINE1
REST CHEA	4.1E-01	5.6E-01	2.09	1.86	CPNE9
RUNX1 CHEA	4.1E-01	5.6E-01	2.07	1.82	SERPINE1
TP63 CHEA	4.4E-01	5.6E-01	1.90	1.56	SLC7A11
USF1 ENCODE	4.5E-01	5.6E-01	1.87	1.51	HMOX1
UBTF ENCODE	4.9E-01	5.9E-01	1.61	1.14	GCLM
CTCF ENCODE	5.3E-01	6.1E-01	1.45	0.93	GCLM
MAX ENCODE	5.8E-01	6.5E-01	1.24	0.67	SERPINE1
ZBTB7A ENCODE	6.0E-01	6.5E-01	1.17	0.59	OSGIN1
ATF2 ENCODE	7.1E-01	7.3E-01	0.86	0.30	SLC7A11
BRCA1 ENCODE	7.5E-01	7.5E-01	0.74	0.21	SLC7A11
